# A web application for gene-based queries of CaeNDR RNA-seq data

**DOI:** 10.17912/micropub.biology.001194

**Published:** 2024-06-05

**Authors:** Avery Davis Bell, Annalise B Paaby

**Affiliations:** 1 School of Biological Sciences, Georgia Institute of Technology, Atlanta, Georgia, United States

## Abstract

Variation in gene expression is a feature of all living systems and has recently been characterized extensively among wild strains of the model organism *Caenorhabditis elegans.* To enable researchers to query gene expression and gene expression variation at any gene of interest, we have created a user-friendly web application that shares RNA-seq transcription data for 208 wild *C. elegans* strains generated by the *Caenorhabditis* Natural Diversity Resource (CaeNDR). Here, we describe the features of the web application and the details of the data and data processing underlying it. We hope that this website, wildworm.biosci.gatech.edu/cendrexp/ , will help *C. elegans* researchers better understand their favorite genes and strains.

**Figure 1. Gene-specific expression quantifications across 208 C. elegans strains and for individual user-selected strains from our new web application:**
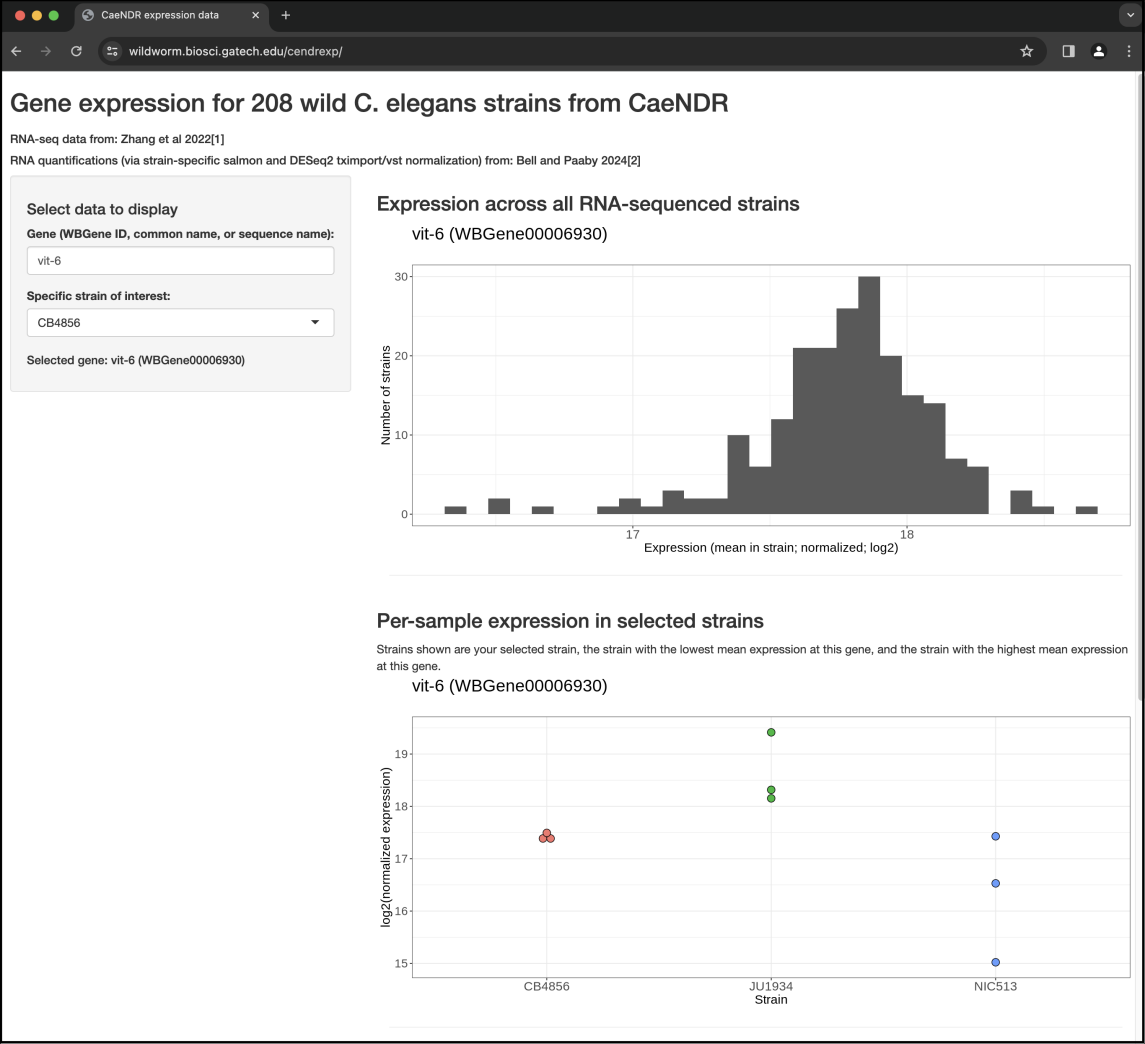
Example results from the web application wildworm.biosci.gatech.edu/cendrexp/ for the user-selected gene *vit-6* (entered into textbox in side panel) and user-selected strain of interest (chosen from dropdown menu in side panel). Top: histogram of the normalized expression for this gene across all 208 *C. elegans* strains. Bottom: Dot plot showing expression estimates for each sequenced sample of the user-selected strain, the strain with the highest mean expression at this gene, and the strain with the lowest mean expression at this gene. The web application has more content accessible by scrolling down (see scrollbar at right of picture, description in main text).

## Description

The recent work of Zhang and colleagues (Zhang et al., 2022) offers a major contribution to the resource-rich model system *C. elegans* : genome-wide RNA sequencing and associated expression quantitative trait locus mapping for over 200 wild strains from the *Caenorhabditis* Natural Diversity Resource (CaeNDR, Cook et al. *,* 2017; Crombie et al. *,* 2024). The sequenced strains span the ranges of global and genetic diversity of the species, and variation in gene expression across the strains is associated with organism-level phenotypes and may be used to improve genetic mapping of complex traits in this system (Zhang et al *.,* 2022). The expression data are publicly available via NCBI, but these data files are large and difficult to query, so expression estimates and expression variation across strains are difficult to ascertain directly. Moreover, the expression estimates are at the transcript level; some researchers may prefer gene-level estimates. While these expression data are in the process of being integrated into the CaeNDR website (Erik Andersen, personal communication), we offer a user-friendly web application to query the expression of any given gene across the population and for specific strains.

The web app, written in Shiny ( https://shiny.posit.co/ ), is available at wildworm.biosci.gatech.edu/cendrexp/ . The user specifies the gene of interest by WBGene ID (preferred), common gene name, or sequence name, and the app displays its normalized expression across all 208 strains in the dataset. The user may also specify a specific strain of interest, to display expression estimates from each experimental sample for this focal strain as well as from those strains with the highest and lowest expression for this gene ( Figure 1 ). Specifically, the app displays normalized expression quantification estimates derived from pseudo-alignment to strain-specific transcriptomes, followed by combining transcript-level expression into gene-level expression, length- and library-size normalization, and variance-stabilizing transformation (see Methods).

Additionally, the app displays further information about the user-selected gene and user-selected strain to aid in interpretation. This information includes the physical chromosomal location of the gene in WS286 coordinates (Davis et al., 2022), whether the gene is classified as hypervariable (formerly termed hyperdivergent) in the focal strain in CaeNDR (Lee et al., 2021), and whether the strain is hypervariable in any CaeNDR strain in the 20220216 release (Lee et al., 2021). The app further reports numerical information about the gene’s expression level, including its minimum expression in any strain, its maximum expression in any strain, the point estimate of expression in the user’s chosen strain, and the expression rank of the user’s chosen strain.

We hope that this web application will be useful for those interested in gene expression phenotypes in *C. elegans* and that it will aid in research efforts across the community. We also note that additional apps, which offer similar gene-based queries of genome-wide gene expression in *C. elegans* , are available at wildworm.biosci.gatech.edu ; those datasets include differential expression across five strains in normal and embryonic knock-down conditions (Bell et al., 2023) , and differential and allele-specific expression for seven strains and their F1 progeny.

## Methods

All workflows and scripts used in the processing of the expression data for this app, as well as the app data and app script itself, are available at https://github.com/paabylab/proccaendrexp . We also briefly describe the methods contained therein here.

Sequence data were downloaded from the NCBI’s Sequence Read Archive (SRA) accession code PRJNA669810 as FASTQ files using the SRAToolKit (v3.0.5). Gene expression estimates and sample information were downloaded from the NCBI’s Gene Expression Omnibus, accession code GSE186719.

For each strain for which RNA-seq data was available, we generated strain-specific transcriptomes by inserting the strain’s variants into the reference genome. Specifically, we used variants from CaeNDR (Cook et al., 2017; Crombie et al., 2024) 20220216 release hard-filter isotype VCF and used g2gtools ( http://churchill-lab.github.io/g2gtools/ ) to adjust the WS286 N2 reference transcriptome (from WormBase, Davis et al., 2022) to be a strain-specific transcriptome for each strain, as described in detail previously (Bell et al., 2023) and using software and tools listed in the reagent table.

We used Salmon (v1.4.0) (Patro et al., 2017) to determine transcript-level quantification estimates genome-wide by first generating salmon indexes of the strain-specific transcriptomes with command *salmon index -k 31 -keepDuplicates* (all others default; no decoy was used) followed by quantification with *salmon quant* . To get per-sample and per-strain gene-specific quantification estimates, salmon quantifications were imported into DESeq2 (v1.42.0) (Love et al., 2014) using tximport (v1.20.0) (Soneson et al., 2015). We further normalized these gene counts by incorporating library size, transcript length, and variance reduction factors using DESeq2’s variance-stabilizing transformation (function *vst* ).

These gene expression abundance estimates were made public using the R (R Core Team 2023) /RStudio-based Shiny ( https://shiny.posit.co/ ) and hosted online by the College of Science’s Academic & Research Computing Services at the Georgia Institute of Technology.

## Reagents

**Table:** 

**Software/Tool**	**Website(s)**	**Version used**	**Publication reference**
SRAToolkit	https://github.com/ncbi/sra-tools/wiki	v3.0.5	-
g2gtools	https://github.com/churchill-lab/g2gtools ; http://churchill-lab.github.io/g2gtools/	v0.1.31 (via Conda v4.7.12 with Python v2.7.16)	-
gffread	https://ccb.jhu.edu/software/stringtie/gff.shtml#gffread ; https://github.com/gpertea/gffread	v0.12.7	(Pertea and Pertea 2020)
Trimmomatic	http://www.usadellab.org/cms/?page=trimmomatic ; https://github.com/usadellab/Trimmomatic	v0.3.9	(Bolger et al., 2014)
Salmon	https://combine-lab.github.io/salmon/	v1.4.0	(Patro et al., 2017)
tximport	https://bioconductor.org/packages/release/bioc/html/tximport.html	v1.20.0	(Soneson et al., 2015)
DESeq2	https://bioconductor.org/packages/release/bioc/html/DESeq2.html	v1.42.0	(Love et al., 2014)
shiny	https://shiny.posit.co/ ; https://CRAN.R-project.org/package=shiny	v1.8.0	-
R	https://www.r-project.org/	v4.3.2	-
RStudio	https://www.rstudio.com/ ; https://posit.co/products/open-source/rstudio/	v2023.12.0+369	-
nextflow	https://www.nextflow.io/	v22.10.7	-

## Extended Data

Description: Code for data processing and underlying the app: github zip of release v0.1. Resource Type: Software. DOI: 10.22002/2h1m6-cpc82
